# Bilateral Chylothorax After 13 Years of Dasatinib Therapy in a Patient With Chronic Myeloid Leukaemia: A Case Report

**DOI:** 10.1002/rcr2.70529

**Published:** 2026-03-06

**Authors:** Riku Watanabe, Shin‐ichiro Iwakami, Manami Haba, Yumi Kuroda, Naoko Iwakami, Eri Fujikawa, Kazuhisa Takahashi

**Affiliations:** ^1^ Department of Respiratory Medicine Juntendo University Shizuoka Hospital Izunokuni Shizuoka Japan; ^2^ Department of Respiratory Medicine Juntendo University Graduate School of Medicine Tokyo Japan

**Keywords:** bilateral chylothorax, chronic myeloid leukaemia, dasatinib

## Abstract

A 52‐year‐old man with chronic myeloid leukaemia (CML) who had been receiving dasatinib 100 mg/day for 13 years presented with progressive dyspnea. Chest radiography revealed bilateral pleural effusions, and further evaluation confirmed the diagnosis of chylothorax. Dasatinib‐induced chylothorax was suspected, and discontinuation of the drug led to clinical improvement. Dasatinib is a tyrosine kinase inhibitor (TKI) used for the treatment of CML and Philadelphia chromosome–positive acute lymphoblastic leukaemia (Ph + ALL). Although pleural effusion is a relatively common adverse effect, chylothorax is an extremely rare complication. This case highlights the importance of considering chylothorax as a potential cause of pleural effusion in patients undergoing long‐term dasatinib therapy. In addition, based on the presumed pathophysiological mechanism, drug discontinuation appears to be the most crucial therapeutic approach for dasatinib‐induced chylothorax.

## Graphical abstract

A 52‐year‐old man with chronic myeloid leukaemia developed bilateral chylothorax after 13 years of dasatinib therapy, presenting with progressive dyspnea. Although pleural effusion is a relatively common adverse effect of dasatinib, chylothorax is an extremely rare complication. Based on the presumed pathophysiological mechanism, this case highlights that drug discontinuation is the most crucial therapeutic approach and that chylothorax should be considered in patients with pleural effusion during long‐term dasatinib treatment.

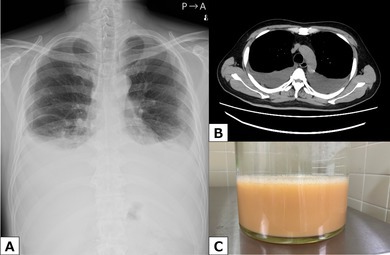

## 1 Introduction

Dasatinib is a tyrosine kinase inhibitor (TKI) used in the treatment of chronic myeloid leukaemia (CML) and Philadelphia chromosome‐positive acute lymphoblastic leukaemia (Ph + ALL). Among its side effects, pleural effusion occurs in approximately 28% of cases and is relatively common [1]. However, chylothorax is a rare complication, with only a limited number of reported cases to date. Here, we report a rare case of bilateral chylothorax that developed after 13 years of treatment with dasatinib at a dose of 100 mg, representing one of the longest durations prior to the onset among the reported cases.

## 2 Case Report

**FIGURE 1:**
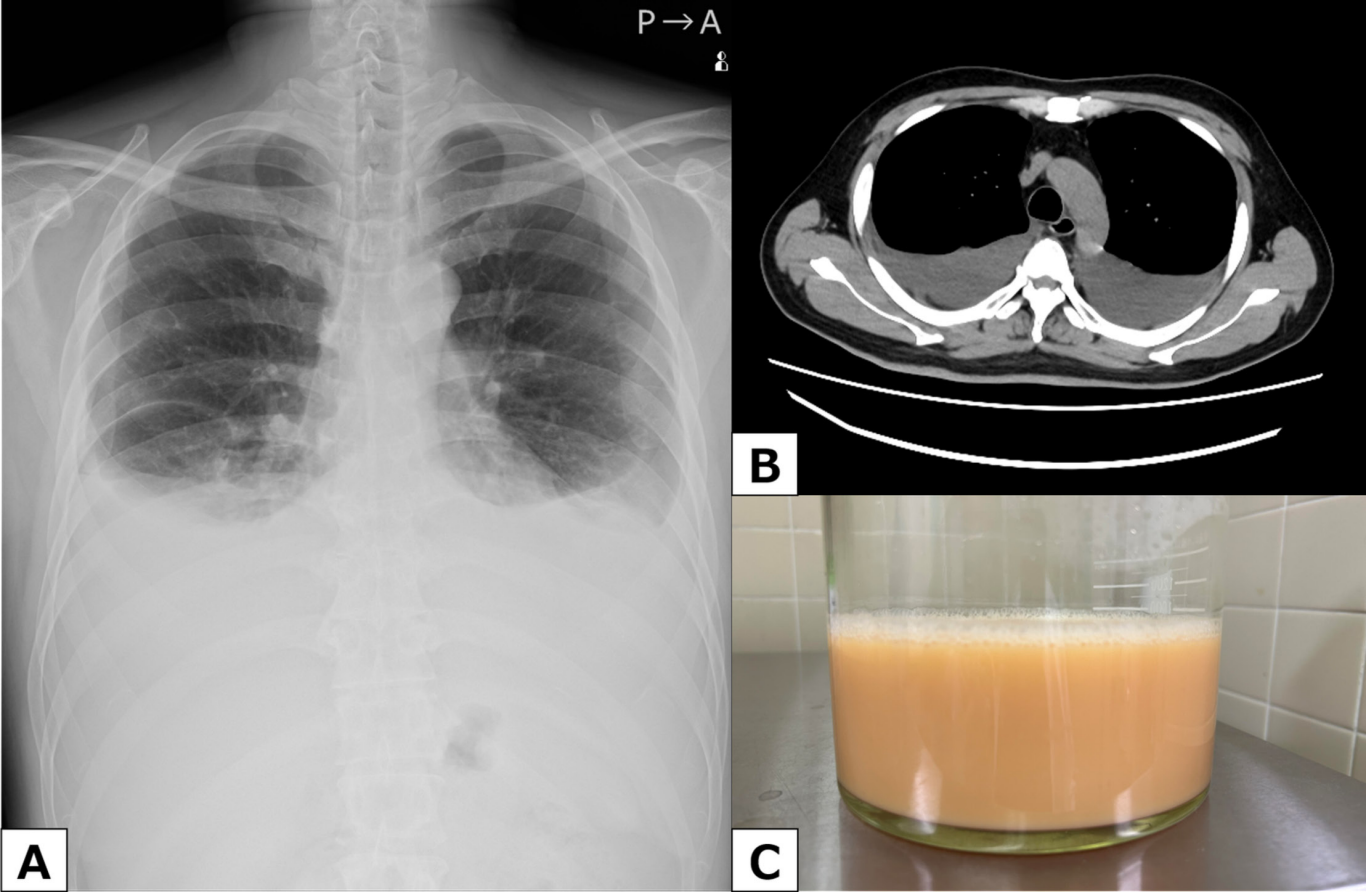
Radiological and thoracentesis findings at initial presentation. (A) Chest radiograph obtained at a routine outpatient visit showing marked bilateral pleural effusions. (B) Computed tomography (CT) also demonstrated marked bilateral pleural effusions, with no evidence of tumours or other structural lesions affecting the thoracic duct. (C) Thoracentesis of the right pleural cavity yielded milky pleural fluid.

A 52‐year‐old man presented with dyspnea. He had been diagnosed with chronic myeloid leukaemia (CML) 13 years earlier and had initiated dasatinib (100 mg/day) at the time of diagnosis. Complete cytogenetic remission was achieved 3 months after the initiation of therapy. Thereafter, he continued dasatinib (100 mg/day) for 13 years and his history was followed for CML at our hospital's Haematology Department. At a routine visit, the subject complained of dyspnea, and underwent chest radiography and computed tomography (CT), which revealed marked bilateral pleural effusions (Figure 1A,B).

There was no evidence of tumours or other lesions affecting the thoracic duct on chest CT findings (Figure 1B). He was referred to our Department for symptom relief and diagnostic evaluation of pleural effusion. Thoracentesis was performed on the right side, which revealed milky and turbid pleural fluid (Figure 1C).

Laboratory analysis of the pleural fluid showed a triglyceride level of 1074 mg/dL, total protein 5 g/dL, LDH 135 U/L, glucose 120 mg/dL, with 28% lymphocytes and 38% macrophages. These findings were consistent with exudative effusion according to Light's criteria and suggested chylothorax. Cytological examination of the pleural fluid revealed no malignant cells.

**FIGURE 2:**
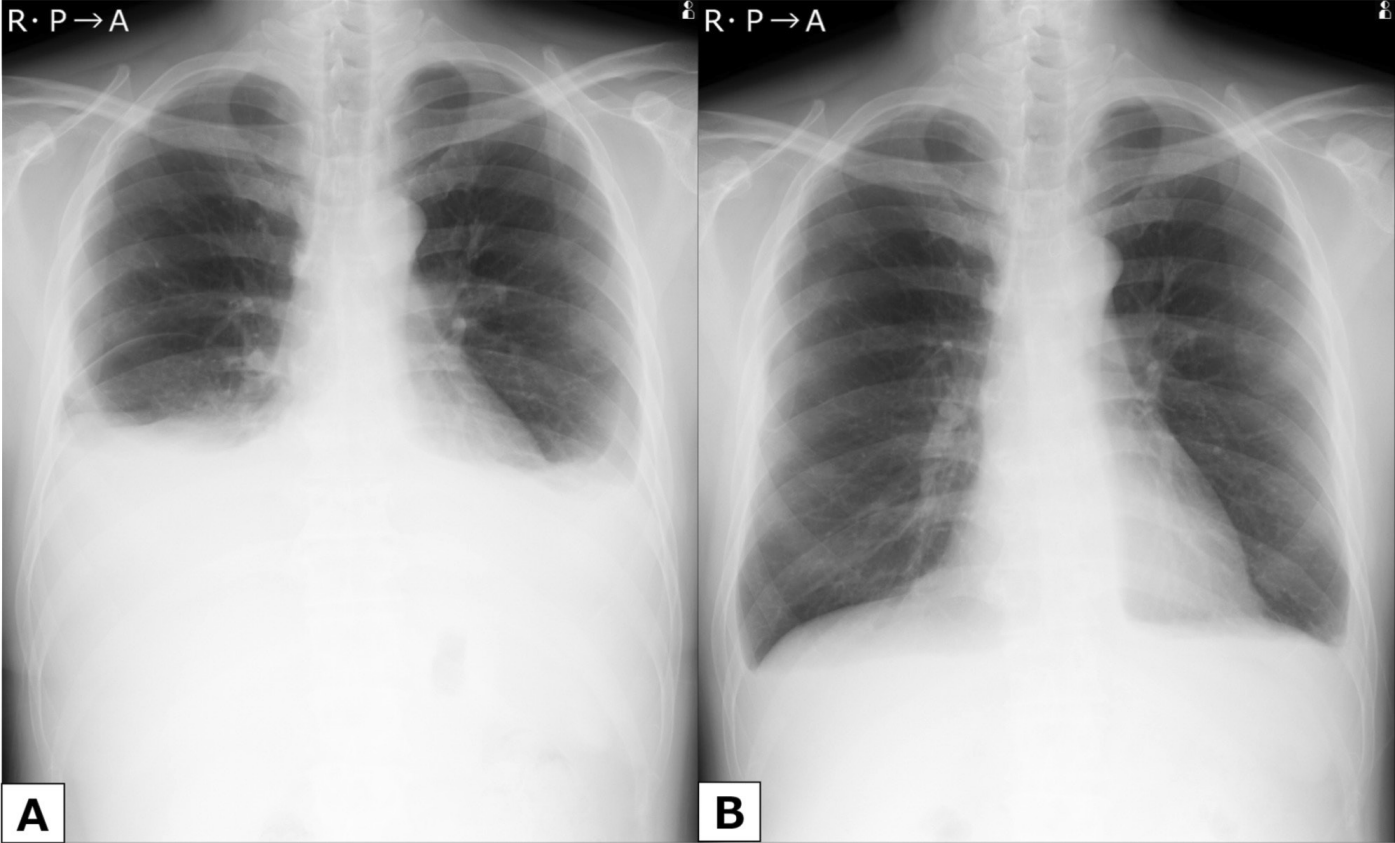
Clinical course of pleural effusion. (A) Chest radiograph at admission, 22 days after the first discontinuation of dasatinib. (B) Final follow‐up chest radiograph obtained 239 days after the initial discontinuation of dasatinib showed marked improvement of bilateral pleural effusions.

Drug‐induced chylothorax was suspected, and dasatinib was discontinued. The patient also began a fat‐restricted diet. Twenty‐two days after cessation of dasatinib, he was hospitalised for pleural effusion management (Figure 2A). On admission, left‐sided thoracentesis was performed and chylothorax was also confirmed on the left side. His symptoms improved after thoracentesis, and he was discharged on hospital day 7. After discharge, dasatinib was reinitiated at a reduced dose of 50 mg/day. However, follow‐up imaging revealed worsening pleural effusion, leading to a second discontinuation of the drug. Following the second cessation, the pleural effusion gradually resolved without further intervention, and the patient was discharged from follow‐up on day 239 after the initial discontinuation (Figure 2B). Importantly, no relapse of CML has been observed, and he has remained stable without resuming dasatinib or initiating any alternative TKIs.

## 3 Discussion

Pleural effusion is a relatively common adverse effect of dasatinib, occurring in approximately 28% of patients [1]. However, dasatinib‐induced chylothorax is an extremely rare complication, with only a limited number of reported cases, and its incidence remains unclear. The present case is notable for the development of chylothorax after long‐term administration of dasatinib for 13 years and for the successful control of the effusion following discontinuation of the drug.

An earlier study reported a median time to onset of pleural effusion of 315 days in patients receiving dasatinib at 100 mg/day [2]. In a review of 16 cases by Pai et al., only three patients developed chylothorax more than 10 years after initiating dasatinib therapy [3] (Table 1). In comparison, the present case represents an exceptionally long duration—13 years—from the initiation of dasatinib to the onset of chylothorax. This underscores the importance of considering chylothorax as a potential cause in patients who develop new‐onset pleural effusion even after more than a decade of dasatinib treatment.

**TABLE 1:** Treatments administered in the 16 cases reported by Pai et al. and in the present case.

No	Age	Sex	Diagnosis	Dosage(mg)	Interval from the use of dasatinib to chylothorax	Laterality	Treatment	Alternative drug
1	71	Male	CML	100	6 months	Bilateral	Thoracentesis, stop dasatinib, but recurrence after dasatinib resumed	N.A.
2	51	Male	CML	100	50 months	Bilateral	Thoracentesis, stop dasatinib, steroid	Nilotinib
3	67	Male	CML	100	8 months after the last dose of dasatinib	Bilateral	Thoracentesis, diuretic, pleurodesis	N.A.
4	73	Female	CML	70	12 months	Right	Thoracentesis, dasatinib changed to bosutinib, then imatinib; diuretic, Japanese herbal medicine “Goreisan”	Imatinib
5	72	Female	CML	100	11 years	Right	Thoracentesis, stop dasatinib	N.A.
6	53	Male	CML	N.A.	14 years	Left	Thoracentesis, stop dasatinib, octreotide	N.A.
7	63	Female	CML	100	4 years	Bilateral	Thoracentesis, dose reduction, then stop dasatinib	Nilotinib
8	11	Boy	CML	50	33 months	Bilateral	Thoracentesis, steroid, diuretic, octreotide, fasting, then stop dasatinib	Nilotinib
9	43	Male	CML	100	123 months	Left	Thoracentesis, stop dasatinib	Bosutinib
10	40	Female	CML	100	40 months	Bilateral	Thoracentesis, steroid, diuretic, stop dasatinib, but recurrence after dasatinib resumed	Nilotinib
11	51	Male	Ph + ALL	140	20 months	Bilateral	Thoracocentesis, stop dasatinib, steroids, diuretics	Imatinib
12	39	Female	CML	50	10 years	Bilateral	Thoracocentesis, stop dasatinib, steroids, diuretics	N.A.
13	69	Male	CML	100	10 months	Bilateral	Thoracentesis, dose reduction, then stop dasatinib	Bosutinib
14	5	Girl	CML	150	14 months	Bilateral	Thoracentesis, stop dasatinib	N.A.
15	71	Female	Ph + ALL	140	2 months	Bilateral	Thoracentesis, dose reduction, steroids, diuretics	N.A.
16	42	Male	CML	40	68 months	Bilateral	Thoracocentesis, stop dasatinib, steroids, diuretics	Nilotinib
Present Case	52	Male	CML	100	13 years	Bilateral	Thoracocentesis, stop dasatinib	N.A.

*Note:* In all cases, symptoms ultimately improved following treatments such as thoracentesis, corticosteroid therapy, diuretic therapy, and discontinuation of dasatinib. In four cases reported by Pai et al. and in the present case, chylothorax recurrence occurred after dasatinib was restarted, suggesting dasatinib as the causative agent and leading to eventual discontinuation of the drug. In addition, nine cases received alternative therapy with other tyrosine kinase inhibitors.

Abbreviations: CML = chronic myeloid leukaemia; N.A. = not applicable; Ph + ALL = Philadelphia chromosome‐positive acute lymphocytic leukaemia.

It has been suggested that the pathogenesis of dasatinib‐induced chylothorax involves the inhibition of platelet‐derived growth factor receptor beta (PDGFR‐β) by dasatinib, which may affect angiogenesis and lymphangiogenesis, ultimately leading to the development of chylothorax [4]. Furthermore, in vivo and in vitro studies by Phan et al. demonstrated that dasatinib alters the distribution of vascular endothelial (VE)‐cadherin and β‐catenin in human pulmonary lymphatic endothelial cells (HPLECs), disrupting intercellular adhesion and increasing endothelial permeability [5]. These structural and functional changes may contribute to the development of chylothorax.

Importantly, Phan et al. also reported that these endothelial alterations were reversible upon discontinuation of dasatinib [5]. In the present case, a similar clinical course was observed: the chylothorax improved after dasatinib cessation, worsened upon rechallenge at a reduced dose of 50 mg/day, and improved again after the second discontinuation. These clinical findings are consistent with the in vitro observations and suggest that reversibility is a characteristic feature of dasatinib‐induced chylothorax.

In this case, although dasatinib was restarted at a reduced dose after initial improvement, pleural effusion worsened again, necessitating a second withdrawal. Similar cases have been reported in which chylothorax recurred or worsened after dose reduction, ultimately requiring permanent discontinuation of dasatinib [3] (Table 1). These findings suggest that dose reduction alone may be insufficient for the resolution of chylothorax, and complete cessation of the drug should be considered.

This case highlights that chylothorax can occur even after prolonged dasatinib administration, and that the underlying mechanism may be reversible, with drug discontinuation being the most critical component of management.

In conclusion, this case taught us several important lessons. Bilateral chylothorax can develop even after more than 10 years of long‐term dasatinib therapy. Furthermore, based on pathophysiological considerations, discontinuation of dasatinib is the most important therapeutic intervention for dasatinib‐induced chylothorax.

## Author Contributions

Riku Watanabe, Shin‐ichiro Iwakami, Manami Haba, Yumi Kuroda, Naoko Iwakami and Kazuhisa Takahashi reviewed the clinical data of this case. Riku Watanabe, Shin‐ichiro Iwakami equally contributed to writing this manuscript. Shin‐ichiro Iwakami and Eri Fujikawa were involved with the management of the patient. All authors have read and approved the final manuscript.

## Funding

The authors have nothing to report.

## Consent

The authors declare that written informed consent was obtained for the publication of this manuscript and accompanying images and attest that the form used to obtain consent from the patient complies with the Journal requirements as outlined in the author guidelines.

## Conflicts of Interest

Kazuhisa Takahashi is an Editorial Board member of Respirology Case Reports and a co‐author of this article. He was excluded from all editorial decision‐making related to the acceptance of this article for publication. The other authors have no conflicts of interest.

## Data Availability

The data that supports the findings of this study are available in this article.
